# Engineering Anisotropic Muscle Tissue using Acoustic Cell Patterning

**DOI:** 10.1002/adma.201802649

**Published:** 2018-09-12

**Authors:** James P. K. Armstrong, Jennifer L. Puetzer, Andrea Serio, Anne Géraldine Guex, Michaella Kapnisi, Alexandre Breant, Yifan Zong, Valentine Assal, Stacey C. Skaalure, Oisín King, Tara Murty, Christoph Meinert, Amanda C. Franklin, Philip G. Bassindale, Madeleine K. Nichols, Cesare M. Terracciano, Dietmar W. Hutmacher, Bruce W. Drinkwater, Travis J. Klein, Adam W. Perriman, Molly M. Stevens

**Affiliations:** ^1^ Department of Materials Department of Bioengineering, and Institute for Biomedical Engineering Imperial College London London SW7 2AZ UK; ^2^ National Heart and Lung Institute Imperial College London London W12 0NN UK; ^3^ School of Engineering and Applied Sciences Harvard University Cambridge MA 02138 USA; ^4^ Institute of Health and Biomedical Innovation Queensland University of Technology Brisbane Queensland 4059 Australia; ^5^ Australian Research Council Training Centre in Additive Biomanufacturing Queensland University of Technology Brisbane Queensland 4059 Australia; ^6^ Department of Mechanical Engineering University of Bristol Bristol BS8 1TR UK; ^7^ Bristol Centre for Functional Nanomaterials HH Wills Laboratory Tyndall Avenue Bristol BS8 1TL UK; ^8^ Centre for Organized Matter Chemistry and Centre for Protolife Research School of Chemistry University of Bristol Bristol BS8 1TS UK; ^9^ School of Cellular and Molecular Medicine University of Bristol Bristol BS8 1TL UK

**Keywords:** acoustic, muscle, patterning, tissue engineering, ultrasound standing waves

## Abstract

Tissue engineering has offered unique opportunities for disease modeling and regenerative medicine; however, the success of these strategies is dependent on faithful reproduction of native cellular organization. Here, it is reported that ultrasound standing waves can be used to organize myoblast populations in material systems for the engineering of aligned muscle tissue constructs. Patterned muscle engineered using type I collagen hydrogels exhibits significant anisotropy in tensile strength, and under mechanical constraint, produced microscale alignment on a cell and fiber level. Moreover, acoustic patterning of myoblasts in gelatin methacryloyl hydrogels significantly enhances myofibrillogenesis and promotes the formation of muscle fibers containing aligned bundles of myotubes, with a width of 120–150 µm and a spacing of 180–220 µm. The ability to remotely pattern fibers of aligned myotubes without any material cues or complex fabrication procedures represents a significant advance in the field of muscle tissue engineering. In general, these results are the first instance of engineered cell fibers formed from the differentiation of acoustically patterned cells. It is anticipated that this versatile methodology can be applied to many complex tissue morphologies, with broader relevance for spatially organized cell cultures, organoid development, and bioelectronics.

The majority of bioengineering strategies use materials with isotropic cell distribution to produce homogenous tissue structures. These engineered tissues, however, generally lack the microscale cellular organization necessary for coordinated mechanical function, biological maturation, and in vivo integration.^1^ Bioprinting offers promise for the assembly of 3D cell networks, however, fabrication of detailed physiological architectures is currently limited by resolution (≈180 µm).^2^ Alternatively, material cues can be used to generate aligned structures at single cell resolution, however, each different pattern requires a new mask, mold, or scaffold.^3^, ^4^, ^5^ Many of these limitations can be addressed by acoustic manipulation, whereby cells are translated toward the static pressure nodes of ultrasound standing waves.^6^ Compared to bioprinting or material cues, acoustic patterning offers simpler fabrication, intermediate feature resolution (≈2–3 cells), and comparable speed (<10 min). Moreover, acoustic patterning enables remote and dynamic manipulation of cell populations within biomaterials, while complex, nonlinear architectures can be generated using hologram‐based acoustics.^7^ To date, 2D and 3D acoustic cell patterning has been used to study biological processes such as neurite guidance,^8^ angiogenesis,^9^ neural differentiation,^10^ and cardiomyocyte beating.^11^, ^12^ Here, we use ultrasound standing waves to direct the assembly of myoblasts in collagen‐based hydrogels, and then stimulate these patterned materials to undergo in situ myogenesis and engineer bundles of aligned myotubes (**Figure** **1**A). This approach, which also resulted in significant tensile anisotropy and enhanced myofibrillogenesis, offers a new platform for the engineering of anisotropic tissue morphologies.

**Figure 1:**
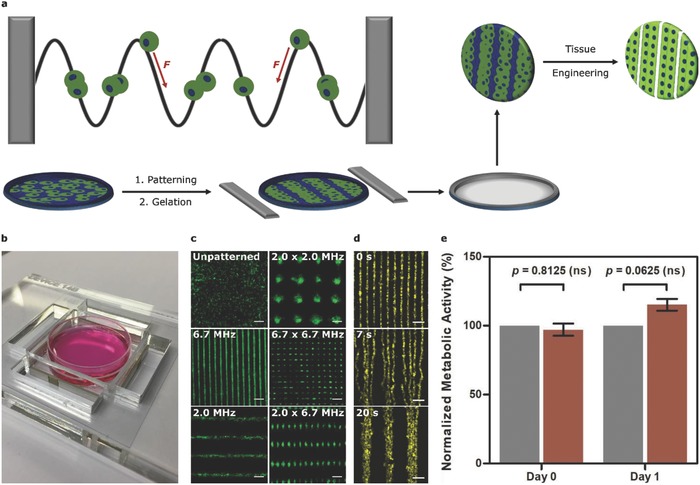
Acoustic cell patterning. a) The acoustic radiation force (*F*) can translate cells toward the pressure nodes of ultrasound standing waves. Controlled gelation processes can be used to pattern cells into materials for tissue engineering. b) The acoustic patterning device, with two pairs of piezotransducers used to generate ultrasound standing waves across a petri dish of cells. c) Wide‐field fluorescence microscopy of myoblasts (green) at 6 × 10^5^ cells mL^−1^ patterned in suspension. Scale bars, 200 µm. d) Time‐lapse microscopy images of myoblasts (yellow) in suspension, first patterned at 6.7 MHz and then switched to a 2.0 MHz field at 3.5 s. Scale bars, 200 µm. e) An alamarBlue assay performed on unexposed myoblasts (gray) or myoblasts exposed for 30 min to 2.0–2.1 MHz ultrasound (red) showed no significant difference in metabolic activity, immediately after exposure or after 1 d of culture. Data shown as mean ± standard deviation, *n* = 5 from five paired exposure experiments, ns = nonsignificant (two‐tailed Wilcoxon matched pairs test).

We designed an acoustic patterning device possessing features compatible with both ultrasound generation and sterile cell culture. We fabricated an acrylic plate with a central cavity to house a 35 mm petri dish containing a suspension of cells. This cavity was flanked by four lead zirconate titanate piezotransducers, which were driven at their resonant frequencies to pattern arrays of C2C12 myoblasts (Figure 1B,C and Figures S1 and S2, Supporting Information). The cell arrays correlated closely with theoretical models and direct empirical measurements of the pressure field, evidence that cells had translated to the nodal planes (Figure S3, Supporting Information). The patterning of myoblasts in cell medium was visualized using confocal fluorescence microscopy, analyzed using a Hough transform pattern recognition algorithm and quantified using a unidirectional patterning index (*S*) calculated from a fast Fourier transform (FFT) (Figure S4, Supporting Information). These analyses revealed a rapid transition from an unordered population with no identifiable features (*S* = 14 ± 5%) to a periodic array of parallel features (S > 90%) in just 30 s. Furthermore, we demonstrated that in situ frequency transitions could be used to dynamically reconfigure patterned cell arrays (Figure 1D).

We tested the compatibility of acoustic patterning for muscle engineering by exposing myoblasts suspended in cell medium to a 2.0–2.1 MHz field for 30 min. This exposure produced no significant detrimental effects upon cell metabolic activity (alamarBlue assay; 0, 1 d), cell proliferation (PicoGreen DNA assay; 1–2 d), myogenic gene expression (MYOG, MRF4; 2–8 d), or muscle protein expression (α‐myosin skeletal fast; 7 d) (Figure 1E and Figures S5 and S6, Supporting Information). We also showed that these field parameters could be used to pattern myoblasts within a range of hydrogels, including agarose, Matrigel, and poly(ethylene glycol) (PEG) norbornene (Figure S7, Supporting Information). However, the material we first selected for muscle engineering was type I collagen; a major component of skeletal muscle^13^ and an established system for myoblast adhesion, survival, and differentiation.^14^ Neutralizing acidified collagen initiated a slow gelation process that we used to encapsulate a thin layer (2–3 cells) of acoustically patterned myoblasts at different material concentrations (1–5 mg mL^−1^) and seeding densities (1–10 × 10^6^ cells mL^−1^) (Figure S8, Supporting Information).

For muscle tissue engineering, we used 3 mg mL^−1^ collagen with a 30 min exposure to an ultrasound standing wave of 0.12 ± 0.2 MPa pressure amplitude to ensure well‐defined patterning, and 3 × 10^6^ myoblasts with a 2.0–2.1 MHz frequency to provide a cell fiber width (≈60–80 µm) that mimicked physiological tissue (40–100 µm).^15^ After gelation, the patterned hydrogels were removed from the field, cultured for 1 d, and then differentiated in myogenic medium for muscle tissue engineering. The collagen effectively maintained the viability of the cells as they shifted from a rounded morphology (*t* = 0 d) into adherent myoblasts (*t* > 1 d) (**Figure** **2**A). Over time, the myoblasts contracted the surrounding matrix yet the patterned configuration was retained throughout. We imaged this process using time‐lapse microscopy and measured a steady reduction in peak‐to‐peak fiber separation from 380 ± 19 to 190 ± 12 µm over 24 h (Figure S9, Supporting Information). Even at day 4, however, the space between adjacent fibers (≈50–70 µm) was still greater than the close‐packed fibers in native muscle.^15^ We exploited the tissue contraction by clamping the patterned collagen during tissue engineering to restrict contraction longitudinally and produce a static load in the direction of the myoblast fibers (Figure 2B,C). These conditions enabled us to engineer tissues with cells individually oriented within the acoustically patterned fibers. This hierarchical structure could not be achieved through clamping alone, with clamped unpatterned tissues exhibiting cell orientation but no bulk organization (Figure S10, Supporting Information).

**Figure 2:**
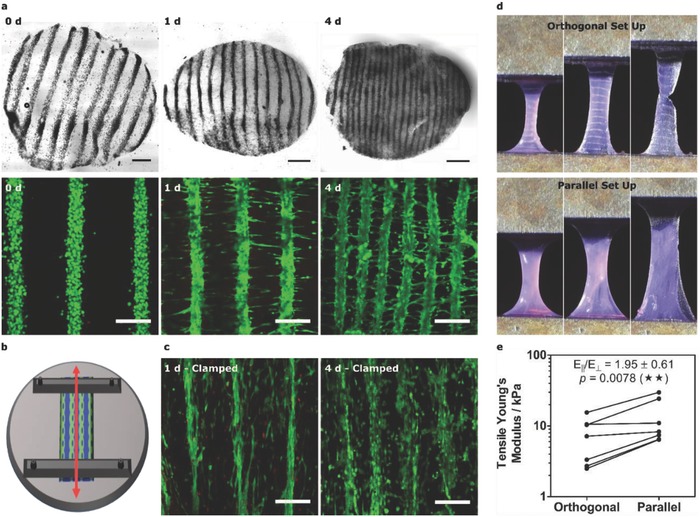
Engineering patterned muscle using collagen. a) Bright‐field and confocal fluorescence microscopy of acoustically patterned myoblasts in 3 mg mL^−1^ collagen. 4 mm diameter biopsy sections, isolated over 4 d, were stained with calcein (green, viable cells) and ethidium homodimer (red, nonviable cells). Bright‐field scale bars, 0.5 mm. Fluorescence scale bars, 200 µm. b) Schematic of mechanical clamping showing how imposed boundary conditions were used to generate static tensile load (red arrow) parallel with the patterned myoblast lines. c) Confocal fluorescence microscopy of the clamped constructs revealed cell‐level orientation and reduced interfiber contraction. Scale bars, 200 µm. d) Mechanical testing was performed with tensile strain applied either orthogonal or parallel with the cell lines. e) The tensile Young's modulus for the orthogonal and parallel configurations. Paired data from seven separate tissues (one‐tailed Wilcoxon matched pairs test), *p* ≤ 0.01 (**).

We evaluated the mechanical anisotropy of the acoustically patterned, unclamped constructs after 1 d in culture, using quasi‐static tensile loading with strain applied either parallel or orthogonal to the patterned cell lines (Figure 2D,E). We measured an 80% increase in tensile Young's modulus for the parallel configuration (*E_‖_* = 13.4 ± 9.6 kPa) compared to the orthogonal setup (*E_Ʇ_* = 7.4 ± 5.0 kPa), the first example in which acoustic patterning has been used to engineer mechanical anisotropy into a cellularized biomaterial. We attributed the tensile anisotropy to the aligned intercellular interactions spanning the length of the construct; for reference, the detachment force required to separate a pair of myoblasts is ≈12 nN after just 10 min of adhesion.^16^ We next evaluated the differentiation state of the tissue after 4 d using quantitative polymerase chain reaction (qPCR) and immunostaining, in which we observed upregulation of myogenic regulatory factors (MYOG, MRF4) and positive staining for contractile proteins (α‐myosin skeletal fast, tropomyosin) (Figure S11, Supporting Information).

Although collagen supported the culture and differentiation of patterned myoblasts, the matrix contraction made it challenging to assess the long‐term processes of myotube formation and tissue maturation. Accordingly, we investigated whether aligned muscle could be engineered using gelatin methacryloyl (GelMA) instead of collagen. We methacrylated ≈88 ± 1% of the lysine residues of gelatin (Figure S12, Supporting Information) to produce GelMA solutions that could be covalently crosslinked in the presence of photoinitiator and ultraviolet light.^17^ We were able to pattern myoblasts in 40 mg mL^−1^ GelMA, the optimal weight fraction for myofibrillogenesis,^18^ using similar field parameters as for collagen (2.0–2.1 MHz, 12 min). Importantly, the covalent linkages, high weight fraction, and amorphous structure of GelMA ensured that the engineered tissue maintained its original size and interfiber separation distance (**Figure** **3**A). These factors enabled unhindered observation of the latter stages of tissue development, most notably, the formation of elongated myotubes at 7 d. These myotubes were viable, present throughout the tissue and generally confined to the original myoblast pattern. Interestingly, the unpatterned controls exhibited negligible myotube formation (Figure S13, Supporting Information), suggesting that acoustic patterning had facilitated myofibrillogenesis. Having already ascertained that field exposure does not affect myotube formation, this enhancement was attributed to the increased cell–cell contact conferred by patterning.

**Figure 3:**
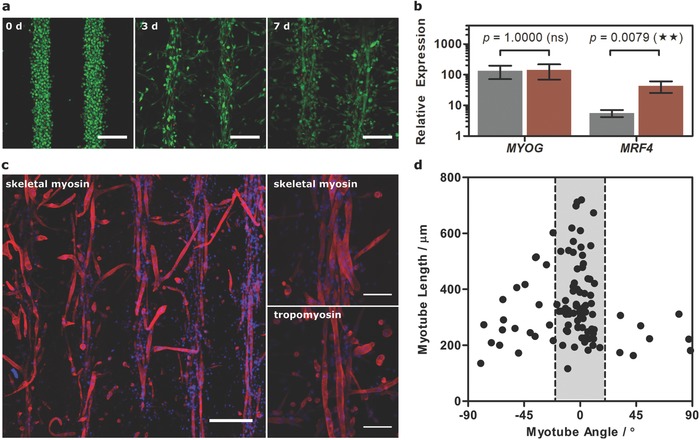
Engineering patterned muscle using GelMA. a) Confocal fluorescence microscopy of acoustically patterned myoblasts in 40 mg mL^−1^ GelMA over 7 d of tissue engineering. Myoblasts were stained with calcein (green, viable cells) and ethidium homodimer (red, nonviable cells). Scale bars, 200 µm. b) Relative expression of MYOG and MRF4 in the unpatterned (gray) and patterned (red) tissues at day 7, compared to undifferentiated myoblasts. Data shown as mean ± standard deviation from five tissues (two‐tailed Mann–Whitney test), *p* ≤ 0.01 (**). c) Immunostaining for α‐myosin skeletal fast and tropomyosin (both red) counterstained with 4′,6‐diamidino‐2‐phenylindole (DAPI, blue, nucleus) in the patterned tissue at day 7. Low‐magnification scale bars, 300 µm. High‐magnification (*z*‐projection over 54 µm) scale bar, 100 µm. d) Myotube length as a function of orientation angle showing that the majority of myotubes were oriented within 20° of the acoustically patterned lines.

qPCR revealed significantly upregulated expression of MYOG and MRF4 for the day 7 patterned tissue, compared to undifferentiated myoblasts (Figure 3B). We observed no significant difference between unpatterned and patterned muscle in the expression of MYOG, which is thought to regulate the early stages of myogenesis.^19^ The patterned tissue did exhibit an eightfold higher expression of MRF4, believed to play a critical role in myotube maturation,^19^ which corroborates the theory that patterning had facilitated myofibrillogenesis. Finally, immunostaining revealed widespread expression of α‐myosin skeletal fast and tropomyosin, with stained myotubes observed predominantly within the acoustically patterned template (Figure 3C). This spatial confinement appeared to direct myoblast fusion, with the majority of myotubes (>70%) oriented within 20° of the acoustically patterned fibers (Figure 3D). Moreover, the oriented myotubes were generally of greater length (0.37 ± 0.14 mm) than the small fraction of orthogonally branching myotubes (0.29 ± 0.12 mm). Finally, we showed that the acoustically patterned fibers at day 7 showed a frequency‐dependent response to pulsed electrical stimulation, evidence of functional patterned myotubes (Figure S14 and Video S1, Supporting Information).

These results demonstrate, for the first time, that aligned cellular fibers can be engineered from a material system bearing acoustically patterned cells. We show that a brief application of ultrasound standing waves (<30 min, 2.0–2.1 MHz) can be used to pattern myoblasts in collagen‐based hydrogels for the engineering of muscle tissue with dense, aligned fibers. This strategy offers great flexibility across different bioengineering protocols, addressing several key limitations facing in vitro muscle formation. Myoblasts patterned in collagen contracted the surrounding matrix to produce high‐density muscle fibers and anisotropic tensile mechanics. By clamping the patterned collagen we could generate, to the best of our knowledge, the first instance of an in vitro engineered tissue exhibiting cell alignment on both an individual cell and population‐wide level. We used a third protocol, with myoblasts patterned in GelMA hydrogels, to significantly enhance myofibrillogenesis and produce tissue constructs with oriented multinucleated myotubes bundled into parallel, aligned muscle fibers. The engineered fiber width (≈120–150 µm) is larger than native tissue (≈40–100 µm)^15^ but comparable to structures formed from micropatterned hydrogels (≈50–330 µm).^3^, ^4^ Overall, we have demonstrated that acoustic patterning can be used to recreate the microscale hierarchical alignment of bundled myotubes without the need for material cues. Future work will focus on macroscale organization, potentially by integrating a vertical standing wave to levitate myoblasts for the assembly of 3D, close‐packed fibers.

## Experimental Section

*Acoustic Cell Patterning*: All reagents from Thermo Fisher Scientific, unless otherwise stated. A 20 V_pp_ driving voltage was supplied by a TG120 20 MHz Function Generator (Aim TTi) to piezotransducers with wrap‐around electrodes (NCE51, Noliac) integrated into the patterning device (see Figure S1, Supporting Information). The impedance was characterized using a TE1000 RF Vector Impedance Analyser (Tomco Technologies). The pressure field was measured using a Fibre‐Optic Hydrophone (Precision Acoustics Ltd.) mounted on a motorized stage, with the output voltage sampled at 100 MHz. The amplitude at 2.0–2.2 MHz was measured (3 × 3 mm, 50 µm pixel size) and the probe calibration was used to calculate the pressure amplitude (0.12 ± 0.02 MPa). Pressure‐field modeling was performed using the Huygens wave theory and the dimensions of the acoustic patterning device. C2C12 myoblasts (ATCC) or RFP/GFP‐expressing C2C12 myoblasts expanded in high glucose Dulbecco's modified Eagle's medium (HG‐DMEM) with 1% (v/v) penicillin/streptomycin and 20% (v/v) fetal bovine serum, were acoustically patterned in 35 mm Petri dishes coated with 2 mL autoclaved 2% (w/v) UltraPure Agarose 1000. Patterning protocols were adapted for each experiment: (1) Myoblasts were patterned for 30 min in 1 mL of neutralized 3 mg mL^−1^ type I collagen in a tissue culture incubator. Collagen extraction, gelation, and clamping were based on published protocols.^20^ For clamped tissue culture, 10 × 35 mm strips of hydrogel, with the patterned lines oriented lengthwise, were secured on a machined platform using stainless steel clamps. (2) Myoblasts were patterned for 12 min in 1 mL of 40 mg mL^−1^ GelMA dissolved in sterile‐filtered 5 mg mL^−1^ Irgacure 2959 (Sigma) in PBS at 37 °C on a Polar Bear Plus hot plate (Cambridge Reactor Design). Ultraviolet irradiation (365 nm, 6 mW cm^−2^) was applied for the final 2 min before washing the hydrogel (2 mL of PBS, then 3 × 2 mL HG‐DMEM). GelMA was synthesized using published protocols^17^ and characterized using an Avance 600 MHz spectrometer (Bruker) and a (2,4,6‐trinitrobenzene sulfonic acid) assay (Sigma). (3) RFP‐myoblasts were patterned for 10 min in 25% (v/v) Matrigel Matrix (Corning) on ice, followed by 5 min gelation at 37 °C. (4) RFP‐myoblasts were patterned for 10 min in 0.5% (w/v) ultralow gelling temperature agarose (Sigma) at 37 °C, followed by 30 min gelation at 25 °C. (5) RFP‐myoblasts were patterned for 2 min in 8% (w/v) 20 kDa eight‐arm PEG norbornene (synthesized using established protocols),^21^ 13.25 mg mL^−1^ 1 kDa PEG dithiol (Sigma), and 0.54 mg mL^−1^ Irgacure 2959, followed by 7 min ultraviolet irradiation (365 nm, 6 mW cm^−2^). (6) Calcein‐stained myoblasts were patterned for 5–10 min in 2 mL of expansion medium with 20 × 10^−3^ m HEPES buffer.

*Tissue Engineering and Analysis*: Collagen and GelMA constructs, including clamped hydrogels, were maintained in expansion medium for 1 d, then differentiated in HG‐DMEM with 1% (v/v) penicillin/streptomycin, 1% (v/v) non‐essential amino acids, 1% (v/v) N‐2, and 20 ng µL^−1^ recombinant human IGF‐1 (PeproTech). Eight analyses were performed: (1) Tensile testing. A 0.1% strain s^−1^ was applied to rectangular tissue biopsies using a 250 g load cell on an Electroforce 5100 (BOSE), with Young's modulus calculated at 0.05–0.15 strain. (2) Immunostaining. Tissues were fixed for 10 min in 4% (v/v) formaldehyde (Sigma), permeabilized for 10 min in 0.5% (v/v) triton X‐100 (Sigma) at 4 °C, blocked overnight in 3% (w/v) bovine serum albumin (BSA, Sigma) in PBS, incubated overnight at 4 °C with 1:4000 anti‐myosin skeletal fast (Sigma) or 1:50 anti‐tropomyosin (Sigma) in 3% BSA/PBS, washed (3 × 5 min 3% BSA/PBS), stained for 2 h with 1:750 anti‐mouse Alexa Fluor 555 in 3% BSA/PBS, washed (3 × 5 min 3% BSA/PBS) and then counterstained for 10 min with DAPI (Sigma). Confocal fluorescence microscopy was used for imaging, with end‐to‐end line measurements of myotubes in GelMA made using FIJI. (3) Gene expression. Tissues were freeze‐thawed at −80 °C with 600 µL TRIzol before mechanical disruption with a TissueLyser II (Qiagen) for 5 min at 15 s^−1^. A chloroform extraction and Direct‐zol RNA Miniprep Kit (Cambridge Bioscience) were used to extract RNA (260:280 > 1.6). A QuantiTect Reverse Transcription Kit (Qiagen) was used to synthesize cDNA (assuming 1:1 conversion). Taqman Probes (CSNK2A2, AP3D1, MYOG, MRF4) and a StepOnePlus Real Time PCR System were used to measure relative expression (ΔΔ*C*_T_) using CSNK2A2 and AP3D1 as endogenous controls. (4) Single timepoint imaging. 4 mm diameter biopsies were isolated at intervals, stained using a LIVE/DEAD kit and imaged using confocal fluorescence microscopy. (5) Contraction imaging. Collagen hydrogels patterned with GFP‐myoblasts were imaged in culture using time‐lapse wide‐field microscopy, with profile plots and separation distances measured at 4 h intervals using FIJI. (6) Electrical pacing. Patterned GelMA tissues at day 7 were stained with fluo‐4 AM dye (Invitrogen) and point stimulated in Tyrode's solution (10 × 10^−3^ m NaCl, 4.5 × 10^−3^ m KCl, 10 × 10^−3^ m glucose, 10 × 10^−3^ m HEPES, 1 × 10^−3^ m MgCl_2_, 1.8 × 10^−3^ m CaCl_2_, pH 7.4) at 37 °C using a MyoPacer Field Stimulator (IonOptix, 20 ms, 40 ± 10 V, 1–4 Hz). Time‐lapse images were captured using wide‐field microscopy (250 fps) and analyzed using MUSCLEMOTION (Open Source).^22^ (7) Patterning imaging. Calcein‐stained myoblasts suspended in expansion medium with 20 × 10^−3^ m HEPES were imaged using time‐lapse confocal fluorescent microscopy, with patterning initiated after 5 s. FIJI and MATLAB R2016b (MathWorks) were used to perform Hough transform, FFT, and time‐resolved FFT. (8) Myoblasts patterned in Matrigel, agarose, PEG norbornene, and expansion medium were imaged using wide‐field microscopy.

*Field Exposure Studies*: 5–6 × 10^6^ myoblasts in expansion medium were exposed to a 2.0–2.1 MHz field for 30 min, with gentle pipetting every 10 min to disrupt the formation of cellular aggregates. An identical setup but without field exposure was used as an unexposed control for four experiments. (1) An alamarBlue assay was performed immediately after field exposure and after 1 d of culture, (2) A Quant‐iT PicoGreen dsDNA Assay was performed after 1 and 2 d of culture, (3) qPCR was performed after 3 d of culture and 2, 4, and 8 d of differentiation, and (4) immunostaining was performed after 3 d of culture and 7 d of differentiation.

## Conflict of Interest

D.W.H., T.J.K., and C.M. are founders and shareholders of GELOMICS PTY LTD, a start‐up company developing and distributing 3D cell culture technology platform. C.M. is also a Director of GELOMICS PTY LTD.

## Supporting information

Supplementary

Supplementary
