# Supplementary material for: Engineering Anisotropic Muscle Tissue using Acoustic Cell Patterning
Source: Adv Mater. 2018 Sep 12;30(43):1802649. doi: 10.1002/adma.201802649 (PMC6386124; doi:10.1002/adma.201802649)
Supplement: Supplementary file 1 — Supplementary [file ADMA-30-na-s002.pdf]

Copyright WILEY-VCH Verlag GmbH & Co. KGaA, 69469 Weinheim, Germany, 2018.

ADVANCED MATERIALS

Supporting Information

for *Adv. Mater.*, DOI: 10.1002/adma.201802649

Engineering Anisotropic Muscle Tissue using Acoustic Cell Patterning

*James P. K. Armstrong, Jennifer L. Puetzer, Andrea Serio, Anne Géraldine Guex, Michaella Kapnisi, Alexandre Breant, Yifan Zong, Valentine Assal, Stacey C. Skaalure, Oisín King, Tara Murty, Christoph Meinert, Amanda C. Franklin, Philip G. Bassindale, Madeleine K. Nichols, Cesare M. Terracciano, Dietmar W. Hutmacher, Bruce W. Drinkwater, Travis J. Klein, Adam W. Perriman, and Molly M. Stevens**

Supplementary Information

Engineering Anisotropic Muscle Tissue using Acoustic Cell Patterning

James PK Armstrong,[1] Jennifer L Puetzer,[1†] Andrea Serio,[1†‡] Anne Geraldine Guex,[1§] Michaella Kapnisi,[1] Alexandre Breant,[1] Yifan Zong,[1] Valentine Assal,[1] Stacey C Skaalure,[1] Oisín King,[2] Tara Murty,[3] Christoph Meinert,[4,5] Amanda C Franklin,[6] Philip G Bassindale,[6,7] Madeleine K Nichols,[6,7,8] Cesare M Terracciano,[2] Dietmar W Hutmacher,[4,5] Bruce W Drinkwater,[6] Travis J Klein,[4,5] Adam W Perriman[9] & Molly M Stevens[1]**

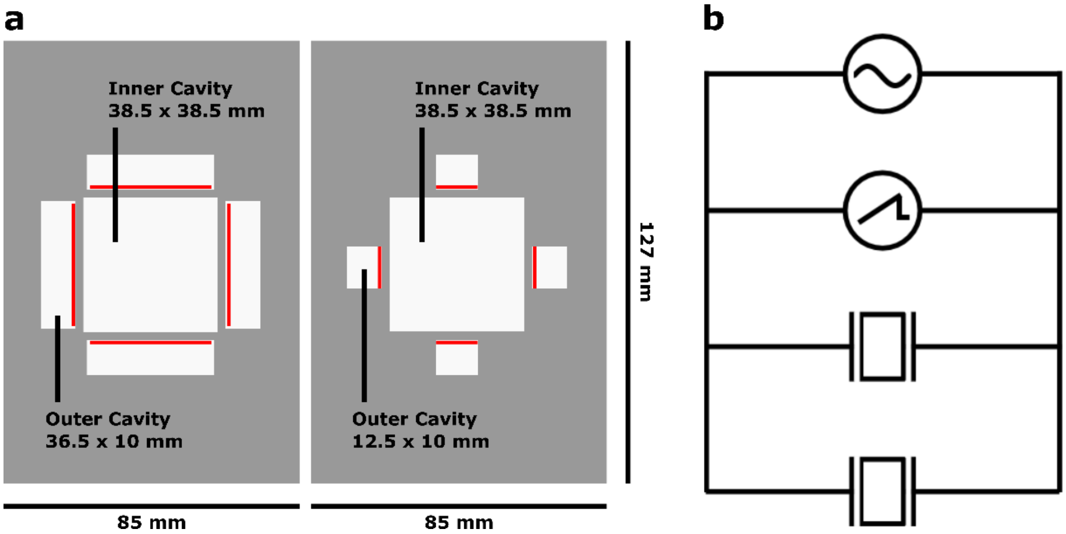

Supplementary Figure 1. Acoustic trapping device design and circuit diagram. (a) The outer dimensions of the plate were designed to match the dimensions of a multiwell plate, allowing ease of imaging, while the central cavity was made to fit a 35-mm petri dish. The four flanking cavities could house piezotransducers of either 12 x 4 x 1 mm or 35 x 4 x 1 mm (shown in red), which were glued and soldered with electrical wire. An acetate sheet glues to the acrylic base allowed the chambers to hold the petri dish and filter-sterilized water. (b) The circuit was composed of a function generator, oscilloscope and a pair of opposing transducers, all connected in parallel. When two pairs of transducers were used, the circuit was replicated with a second function generator.

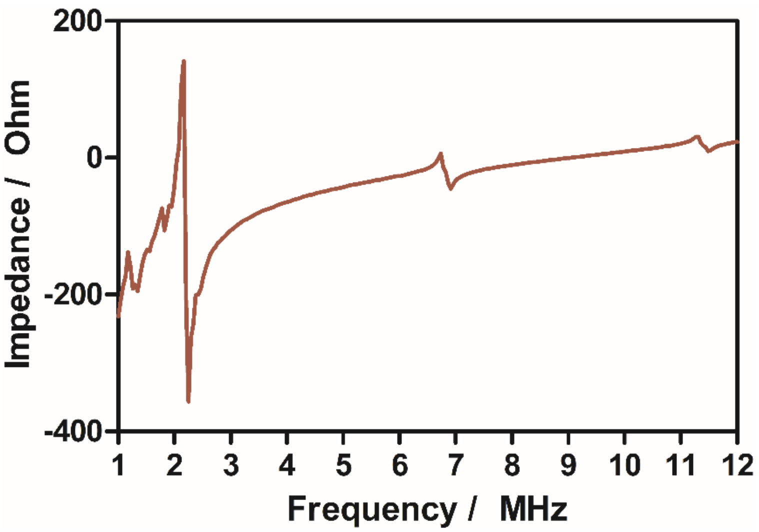

Supplementary Figure 2. Impedance spectroscopy. A frequency sweep was used to measure the impedance of the piezotransducers used to generate the ultrasound standing waves. Resonant frequencies were observed at 2.1-2.3, 6.7-6.9 and 11.3-11.5 MHz. The degree of cell patterning around these values was used to identify the driving frequencies used in the study. For instance, the sharpest patterning was observed at a driving frequency of 2.0-2.1 MHz rather than the exact resonant frequency of 2.1-2.3 MHz.

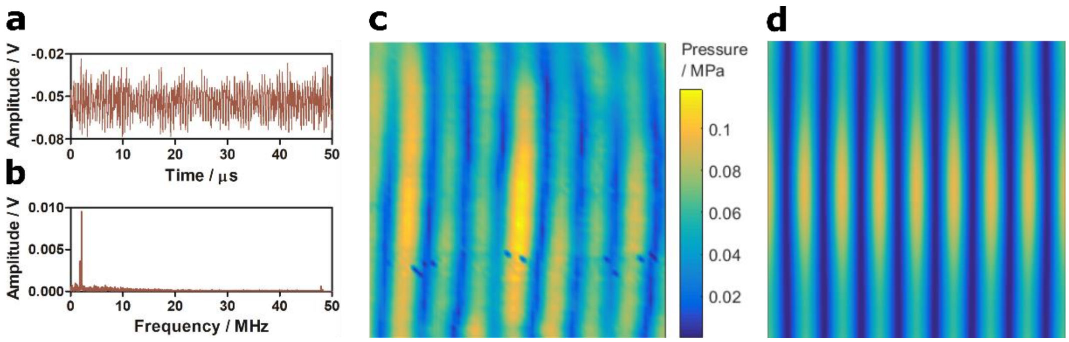

Supplementary Figure 3. Acoustic pressure measurements and modelling. (a) A calibrated fiber optic hydrophone was positioned at the center of the acoustic patterning device, where it was used to record a signal voltage for 5 seconds. (b) The voltage signals were analyzed in the frequency domain to reveal a major peak at the driving frequency (2.1 MHz). (c) The maximum amplitude signal within the frequency band 2.0-2.2 MHz was measured across a 3 x 3 mm scan area, with a raster step size of 50 µm, with the pressure derived from the probe calibration (205.62 mV MPa^{-1}). This revealed the amplitude of the ultrasound standing wave in this region to be 0.12 ± 0.02 MPa. (d) The measured pressure field was comparable to a model of a 1D ultrasound standing wave (2.1 MHz) formed at the center of the acoustic patterning device (3 x 3 mm). This model uses Huygens wave theory to describe the relative pressure field in the acoustic patterning device. The map shows relative values from low pressure (blue) to high pressure (yellow).

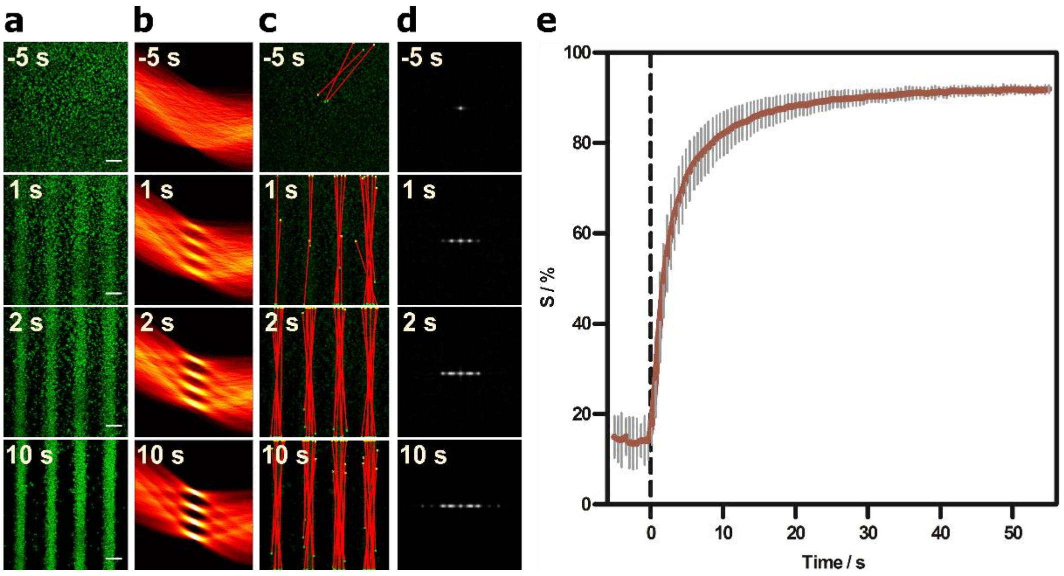

Supplementary Figure 4. Image analysis of acoustic cell patterning. (a) Live-cell confocal fluorescence microscopy was used to capture a time-lapse series of myoblasts (stained green using calcein dye) in culture medium exposed to a 2.0-2.1 MHz ultrasound standing wave. These images were taken prior to field exposure (-5 s) and after 1, 2 and 10 s of acoustic cell patterning. Scale bars, 200 µm. (b) A Hough Transform was used to identify the major features present at the different intervals in the cell patterning process. Application of the acoustic field results in tight nodes centered around 0°, indicating well aligned, parallel features. (c) The dominant extracted features (red lines) corresponded to the acoustically patterned cells in the original micrographs. (d) A 2D FFT algorithm applied to the same images revealed frequencies corresponding to the aligned cell population after 1 s of field exposure. (e) FFT analysis was used to calculate a unidirectional patterning index (S / %) as a function of time (t / s), which showed rapid cell alignment in the first 10 s of exposure to the ultrasound standing wave. Data shown as mean ± standard deviation, n = 10 from ten separate time-lapse sequences.

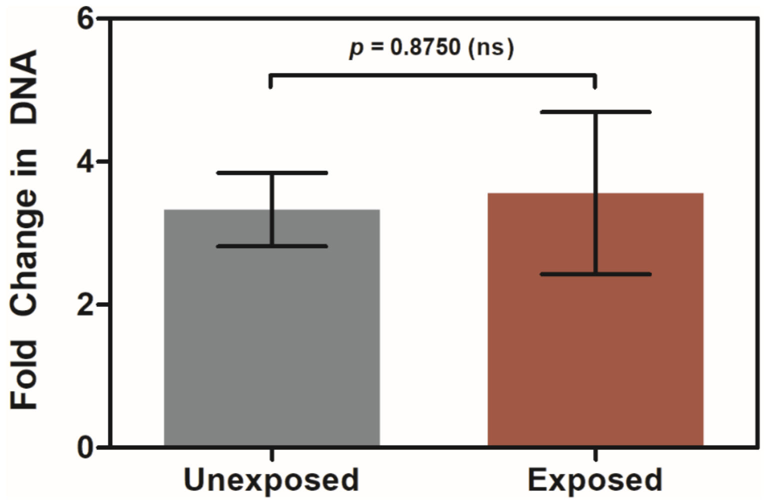

Supplementary Figure 5. Effect of ultrasound upon myoblast proliferation. Myoblasts were exposed for 30 minutes to 2.0-2.1 MHz ultrasound and then cultured, alongside a control of unexposed myoblasts. A PicoGreen assay was used to measure the DNA content of both groups over a 24-hour culture period from day 1 to day 2. This revealed no significant difference in the fold change of DNA between the unexposed myoblasts (gray) and exposed myoblasts (red bars). Data shown as mean ± standard deviation, n = 4 from four paired stimulation experiments, ns = non-significant (two-tailed Wilcoxon Matched Pairs Test).

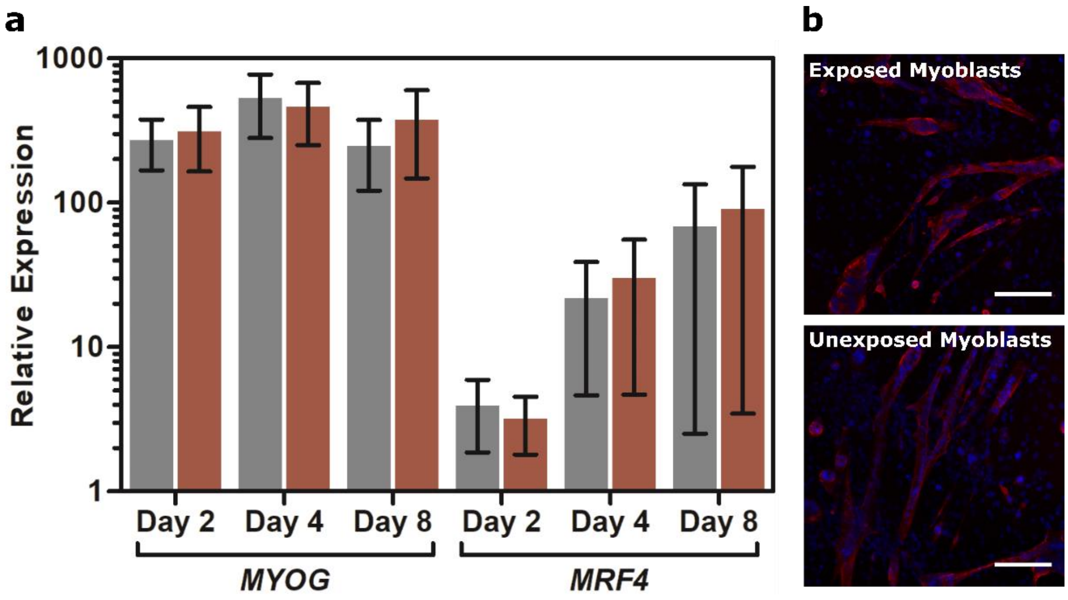

Supplementary Figure 6. Effect of ultrasound upon myoblast differentiation. (a) Myoblasts were exposed to 30 minutes of 2.0-2.1 MHz ultrasound or left unexposed under the same conditions, before undergoing an eight-day course of myogenic differentiation. qPCR was used to quantify the relative expression (ΔΔCt) of the mRNA of MYOG and MRF4 at different time points, compared to undifferentiated myoblasts and normalized to using endogenous controls CSNK2A2 and AP3D1. No significant differences were detected between the samples cultured with unstimulated myoblasts (gray) and stimulated myoblasts (red). Data shown as mean ± standard deviation, n = 3 from three paired stimulation experiments (two-tailed Wilcoxon Matched Pairs Test). (b) Immunostaining of the muscle-specific protein α-myosin skeletal fast (red) and a counterstain of DAPI (blue, nucleus) was used to identify myotubes after seven days of differentiation, in the exposed and unexposed groups. Scale bar, 200 µm.

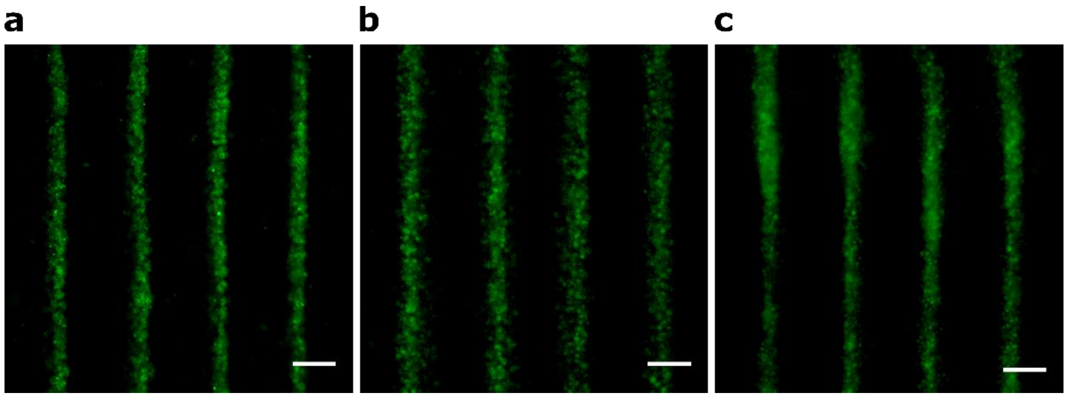

Supplementary Figure 7. Patterning of myoblasts in different biomaterials. Widefield microscopy of myoblasts (green, myoblasts transfected with RFP) patterned within (a) 8% (w/v) 8-arm poly(ethylene glycol) norbornene hydrogel, (b) 0.5% (w/v) agarose and (c) 25% (v/v) Matrigel matrix, all at a density of 3 x 10^6 cells mL^{-1}. Scale bars, 200 µm.

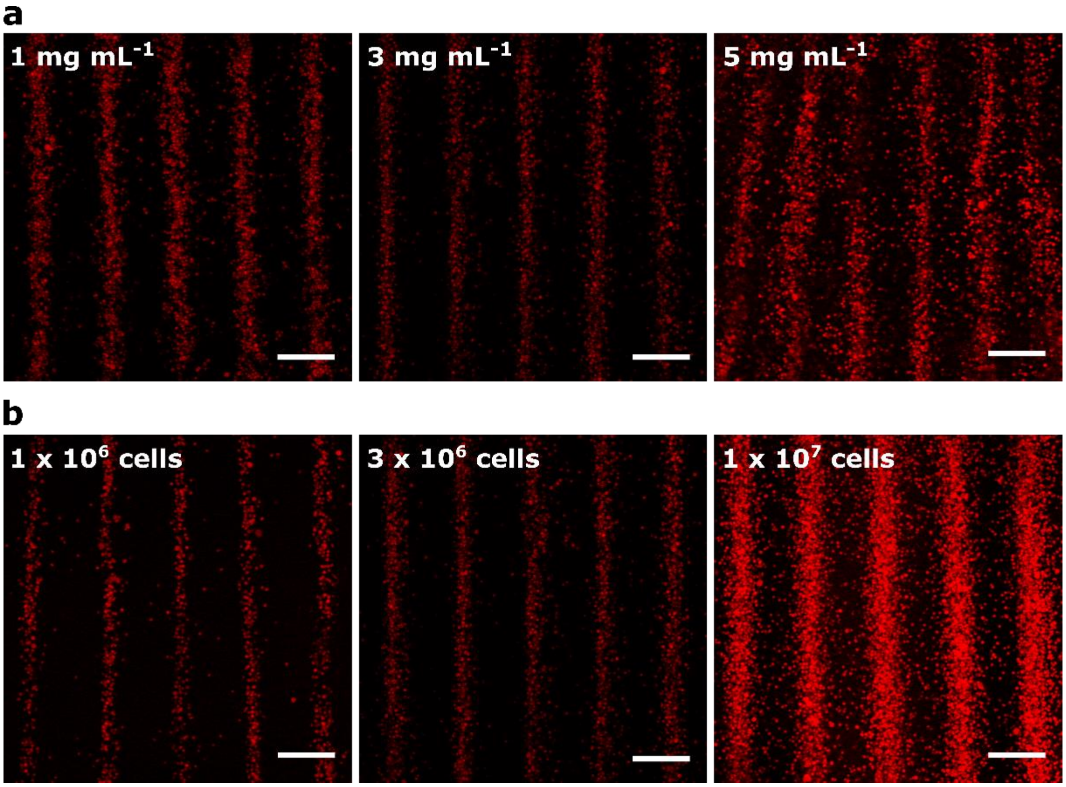

Supplementary Figure 8. Versatility of acoustic cell patterning. (a) 3 x 10^6 myoblasts were patterned into collagen with a gel concentration of between 1-5 mg mL^{-1}. The viscosity impeded patterning at higher concentrations (> 5 mg mL^{-1}), while the hydrogels were fragile at lower concentrations (< 1 mg mL^{-1}). (b) A 3 mg mL^{-1} collagen hydrogel was used to encapsulate patterned myoblasts at cell densities between 1 x 10^6 and 1 x 10^7. The lines were incomplete at lower cell seeding (< 1 x 10^6), while higher cell densities (> 1 x 10^7) produced dense constructs that contracted rapidly in culture. Scale bars, 300 µm.

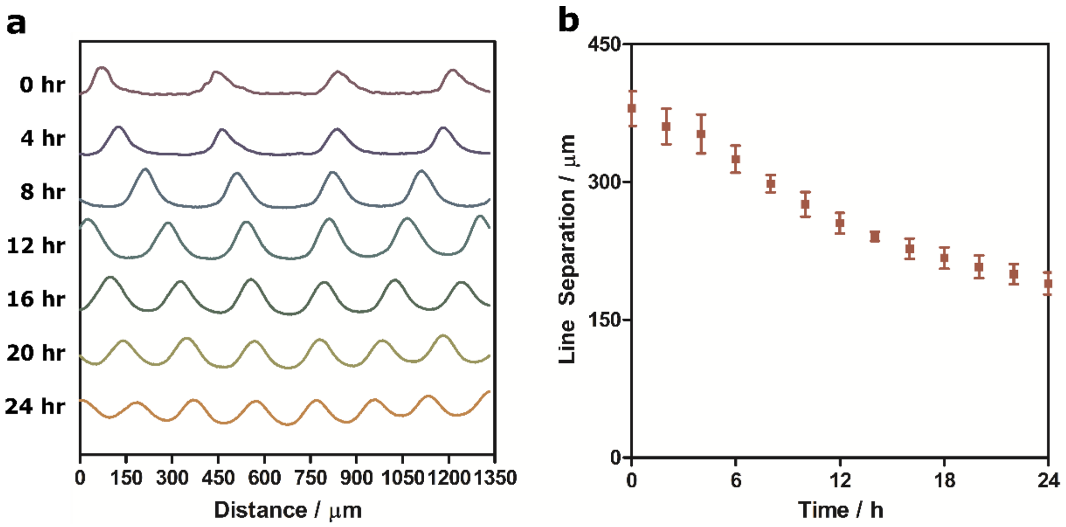

Supplementary Figure 9. Contraction over time. Fluorescence microscopy was used to capture a series of time-lapse images of the contraction of a collagen gel patterned with GFP-C2C12. (a) Fluorescence intensity profiles were plotted at four-hour intervals. (b) The peak-to-peak separation distance was plotted as a function of time, which revealed the gradual rate of contraction over the first 24 hours. Data shown as mean ± standard deviation, n = the number of peaks in the profile.

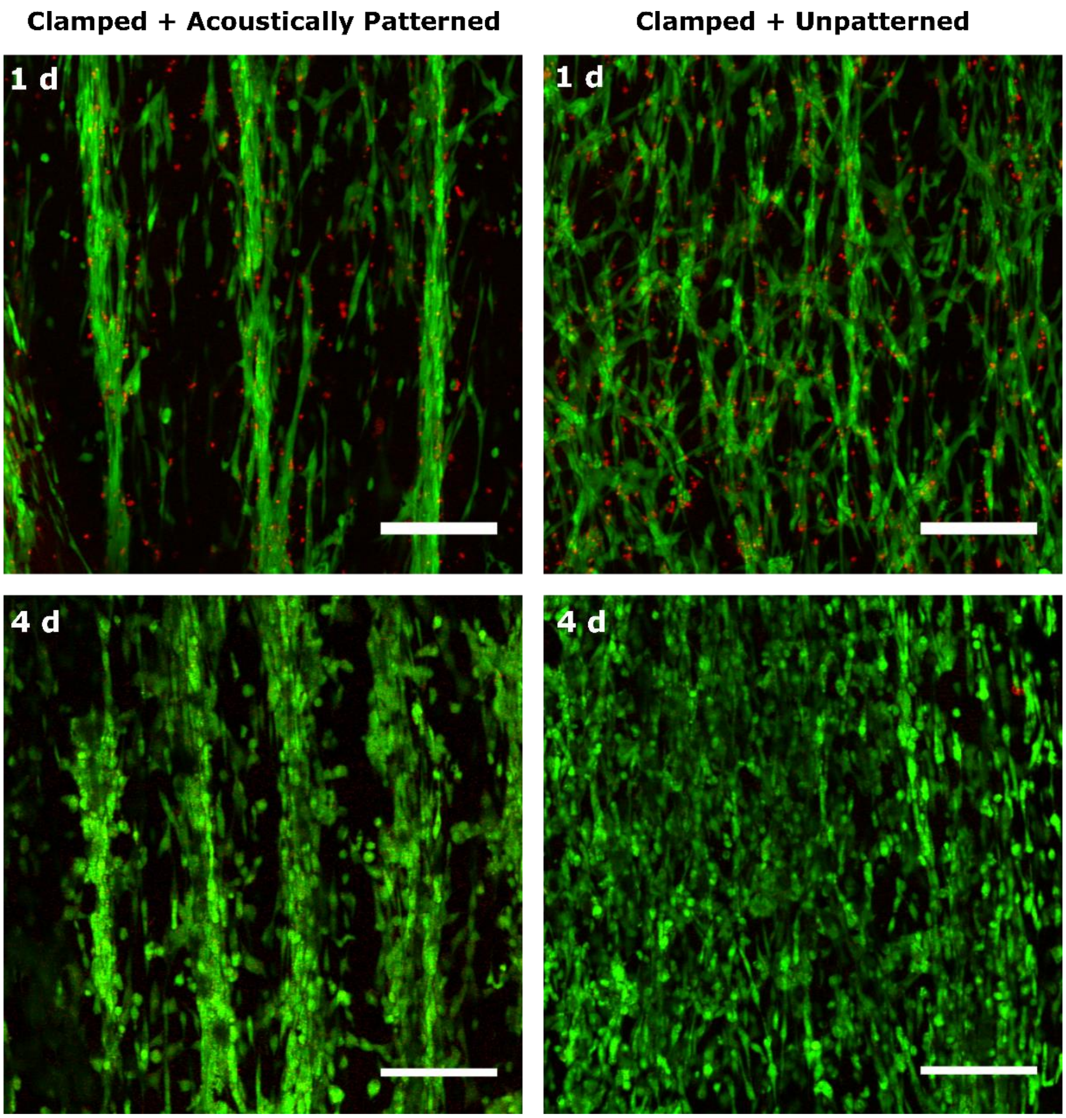

Supplementary Figure 10. Clamping experiment controls. Comparison between clamped constructs that had previously been acoustically patterned and clamped constructs that had not been patterned. Tissue clamping produced cell-level orientation in both cases, however, clamping acoustically-patterned constructs produced both macroscopic and microscopic alignment. The constructs were stained with calcein (green, viable cells) and ethidium homodimer (red, non-viable cells). Scale bars, 200 µm.

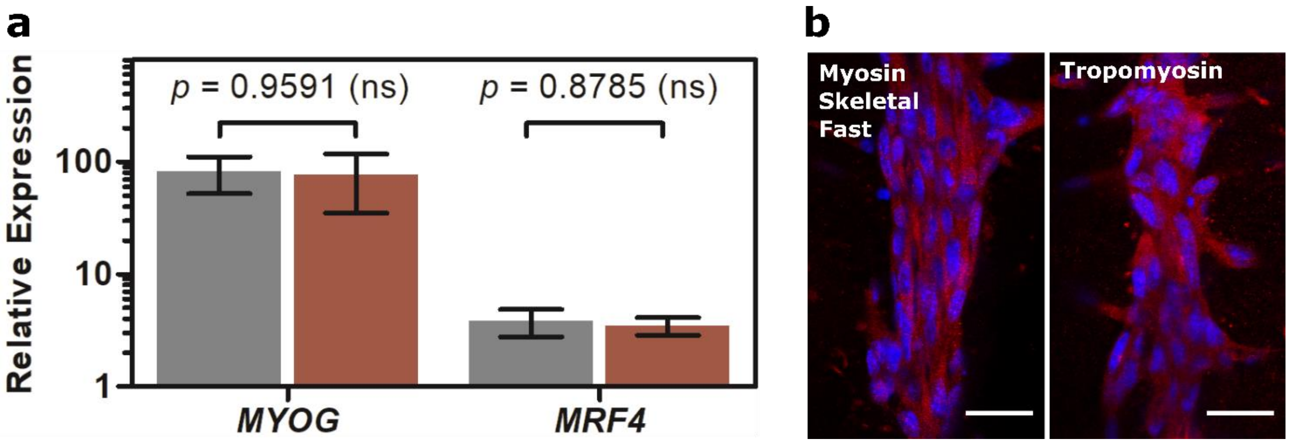

Supplementary Figure 11. Differentiation of acoustically-patterned myoblasts in collagen. (a) qPCR analysis of the relative expression (ΔΔCt) of the mRNA of myogenic regulatory factors MYOG and MRF4 at day 4, compared to undifferentiated myoblasts and normalized using endogenous controls of CSNK2A2 and AP3D1. In all cases, MYOG and MRF4 were upregulated, and there was no significant difference between the relative expression in the patterned tissue (red) and the unpatterned controls (gray). Data shown as mean ± standard deviation, n = 8 from eight separate tissues, ns = non-significant (two-tailed Mann-Whitney Test). (b) Immunostaining of the muscle-specific proteins α-myosin skeletal fast and tropomyosin (both shown in red) and a counterstain of DAPI (blue, nucleus). Scale bars, 20 µm.

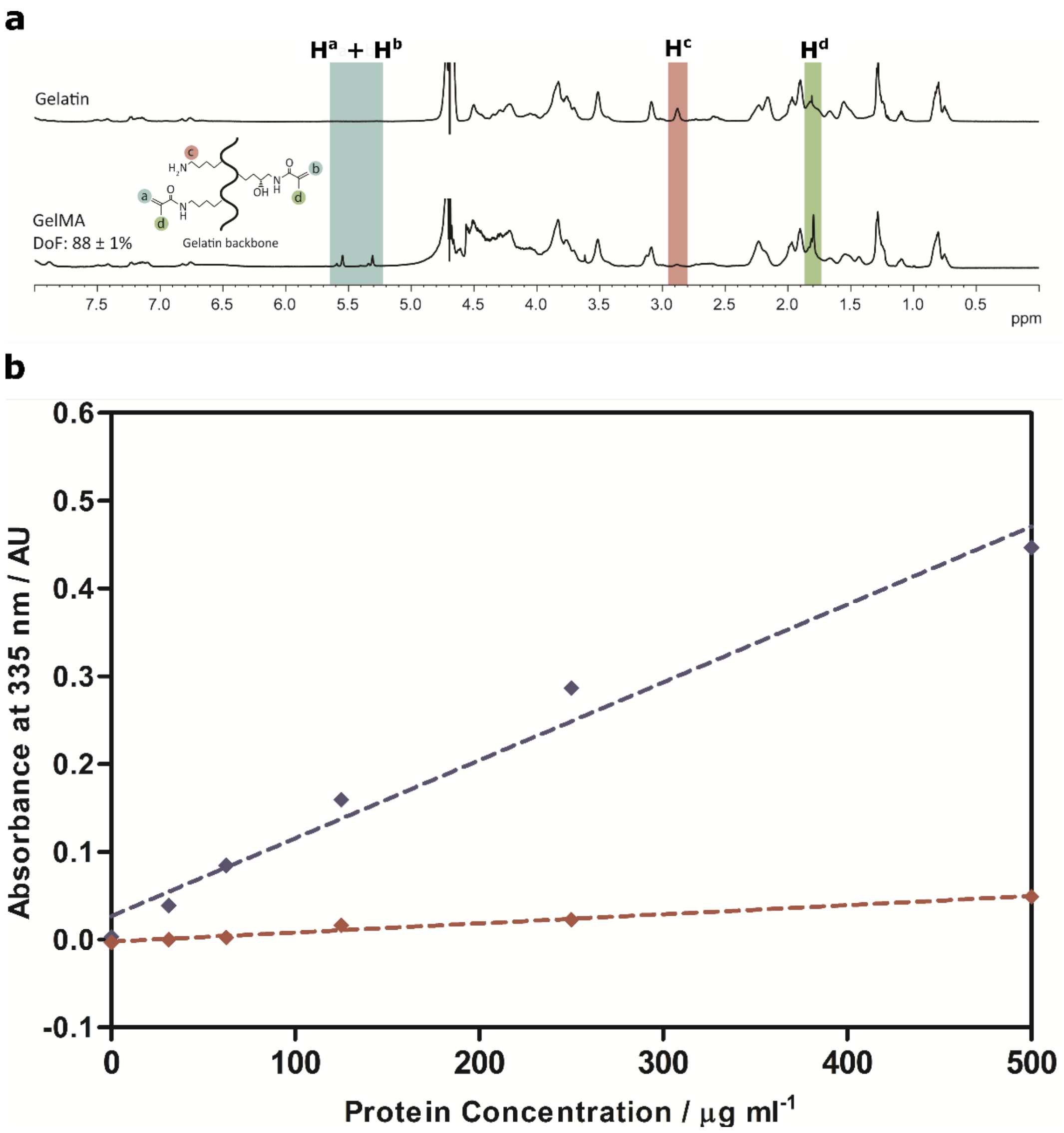

Supplementary Figure 12. Characterization of GelMA. (a) ^{1}H-NMR spectra of gelatin and GelMA. Peaks at ~ 5.6 ppm and ~ 5.3 ppm correspond to acrylic protons (2H) of methacrylamide-functionalized lysine (H^a) and hydroxylysine groups (H^b), peak at ~ 2.7 ppm corresponds to methylene protons (2H) of unreacted lysines (H^c), and peak at ~ 1.8 ppm corresponds to methyl protons (3H) of methacrylamide groups (H^d). (b) TNBS assay of gelatin (blue) and GelMA (red), with the reduced absorbance at 335 nm for GelMA attributed to the reduced number of primary amines post-methacrylation. The gradients of the linear fits were used to calculate a degree of amine functionalization of 88 ± 1%.

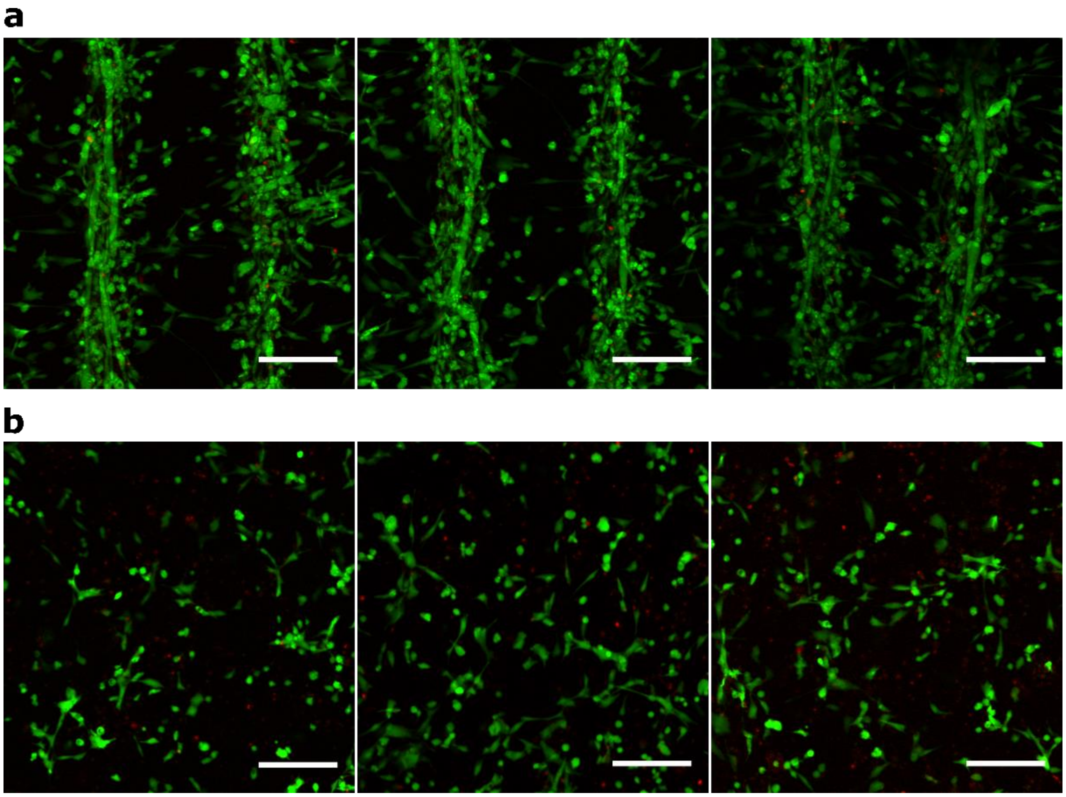

Supplementary Figure 13. Unpatterned controls for day 7 imaging. Live-cell confocal fluorescence microscopy was performed on day 7 muscle tissue engineered from 40 mg mL^{-1} GelMA containing (a) acoustically-patterned myoblasts or (b) unpatterned myoblasts. The tissue was LIVE/DEAD™ stained with calcein (green, viable cells) and ethidium homodimer (red, non-viable cells). The patterned tissue exhibited widespread myotube formation, which was not present in the unpatterned controls. Scale bars, 200 µm.

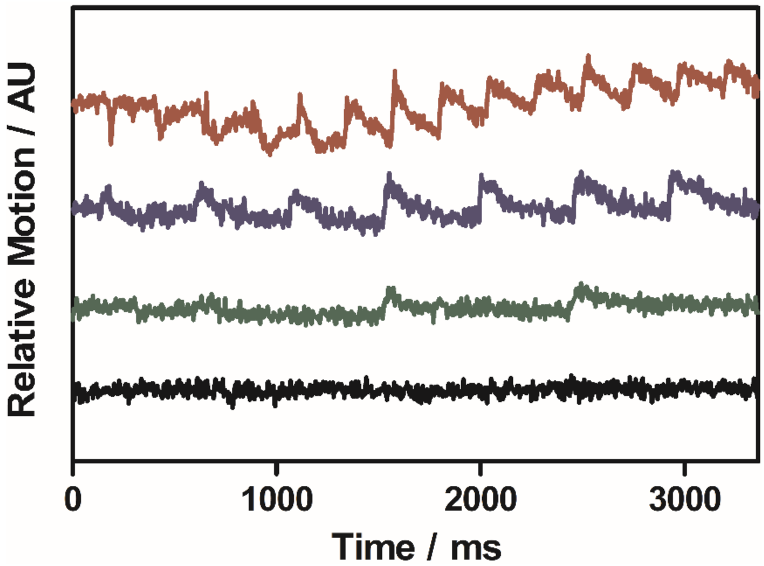

Supplementary Figure 14. Electrical pacing analysis. Time-lapse widefield microscopy was used to record the electrical pacing of an acoustically-patterned day 7 muscle tissue engineered from 40 mg mL^{-1} GelMA (see Supplementary Video 1). Image analysis using MUSCLEMOTION software enabled the relative motion to be quantified for the tissue stimulated using 20 ms pulses at 1 Hz (green trace), 2 Hz (blue trace) and 4 Hz (red trace) frequencies. The stimulated tissue showed a frequency-dependent response that was not present in the unstimulated control (black trace).

Supplementary Video 1. Electrical pacing imaging. Time-lapse widefield microscopy was used to record the electrical pacing of an acoustically-patterned day 7 muscle tissue engineered from 40 mg mL^{-1} GelMA. Pacing was performed using 20 ms pulses at 1, 2 and 4 Hz with an unpaced control. Scale bar, 100 µm.
